# Data on two- and three-dimensional optical coherence tomography guidance for the treatment for the bifurcation lesion

**DOI:** 10.1016/j.dib.2017.12.024

**Published:** 2017-12-14

**Authors:** Ryoji Nagoshi, Takayuki Okamura, Yoshinobu Murasato, Tatsuhiro Fujimura, Masahiro Yamawaki, Shiro Ono, Takeshi Serikawa, Yutaka Hikichi, Fumiaki Nakao, Tomohiro Sakamoto, Toshiro Shinke, Yoichi Kijima, Amane Kozuki, Hiroyuki Shibata, Junya Shite

**Affiliations:** a Department of Cardiology, Osaka Saiseikai Nakatsu Hospital, Japan; b Department of Medicine and Clinical Science, Yamaguchi University Graduate School of Medicine, Japan; c Department of Cardiology, Kyusyu Medical Center, Japan; d Department of Cardiology, Saiseikai Yokohama Eastern Hospital, Japan; e Department of Cardiology, Saiseikai Yamaguchi General Hospital, Japan; f Department of Cardiology, Saiseikai Fukuoka General Hospital, Japan; g Department of Cardiology, Oda Hospital, Japan; h Department of Cardiology, Yamaguchi Central General Hospital, Japan; i Department of Cardiology, Saiseikai Kumamoto General Hospital, Japan; j Department of Cardiology, Kobe University Graduate School of Medicine, Japan

**Keywords:** Optical coherence tomography, Bifurcation stenting, Kissing balloon inflation, Three-dimensional

## Abstract

This article comprised the data related to the research article entitled “Feasibility and usefulness of three-dimensional optical coherence tomography guidance for optimal side branch treatment in coronary bifurcation stenting” (Nagoshi et al., In press) [1].

In this article we reports details about two patterns of guide wire (GW) recrossing position after crossover stenting in bifurcation lesion classified with three-dimensional optical coherence tomography (3D-OCT) (Okamura et al., 2014) [2] and follow-up data about the treatment with percutaneous coronary intervention(PCI) for bifurcation lesion in terms of the two- (2D) or 3D-OCT guidance. Subgroup analysis about differences in the parameters between the proximal and the distal GW recrossing patterns are analyzed here.

**Specifications Table**

**Table:** 

Subject area	*Cardiology*
More specific subject area	*Bifurcation lesion*
Type of data	*Table and figure*
How data was acquired	*Angiography, Optical coherence tomography and survey*
Data format	*Analyzed*
Experimental factors	*The two groups of treatment (2D- and 3D-OCT) were compared*
Experimental features	*Quantitative and qualitative analysis of angiographic and optical findings*
Data source location	*Ten institutes in Japan*
Data accessibility	*Data is with this article*

**Value of the data**

• The data presents two patterns of GW recrossing position after crossover stenting in bifurcation lesion classified with 3D-OCT (Okamura et al., 2014) [1].
• The rate of incomplete stent apposition after kissing balloon inflation with the proximal or with the distal GW recrossing are mentioned.
• Clinical and angiographical follow-up data of bifurcation stenting under 2D- or 3D-OCT guidance are shown in the article.

## 1 Data

Among a total of 150 cases, the guidewire recrossing through the distal cell was achieved in 126 cases(84%). Table 1 shows the data comparing the proximal and the distal guidewire recrossing.

**Table 1:** Baseline, lesion, procedure characteristics and 3D-OCT findings.

	*Proximal Recross*	*Distal Recross*
	(*n*=24)	(*n*=126)
Age (years)	68.0±12.5	70.3±10.0
Male (%)	19 (79)	91 (73)
Clinical presentation		
Stable AP and Silent ischemia (%)	18 (75)	97 (77)
Old MI (%)	2 (8)	11 (9)
ACS (Unstable AP and AMI) (%)	4 (17)	18 (14)
Target vessel		
LMT(%)	3 (13)	26 (21)
LAD-Dx (%)	14 (58)	75 (60)
LCx-OM (%)	4 (17)	17 (13)
RCA PD-PL (%)	3 (13)	8 (6)
Medina classification		
1,1,1	2 (8)	30 (24)
1,1,0	8 (33)	25 (20)
1,0,1	2 (8)	5 (4)
0,1,1	1 (4)	12 (10)
1,0,0	3 (13)	8 (6)
0,1,0	6 (25)	44 (35)
0,0,1	2 (8)	2 (2)
True bifurcations (%)	5 (21)	47 (37)
QCA analysis		
PMV Reference diameter	3.00±0.70	3.15±0.67
DMV Reference diameter	2.34±0.66	2.30±0.49
SB Reference diameter	1.99±0.62	2.31±0.58
PMV % diameter stenosis	37.3±27.7	30.7±26.3
DMV % diameter stenosis	48.5±21.3	47.3±19.7
SB % diameter stenosis	19.4±11.5	25.1±19.8
PMV-DMV Angle	154±18.3	152±22.0
PMV-SB Angle	152±16.2	145±22.7
DMV-SB Angle	53.8±18.5	62.0±22.6
Stent type		
Nobori stent (%)	5 (21)	35 (26)
Promus stent (%)	5 (21)	12 (10)
Resolute Integrity stent (%)	5 (21)	29 (23)
Xience stent (%)	8 (33)	36 (29)
Ultimaster stent (%)	1 (4)	14 (11)
Stent size	2.94±0.46	3.04±0.43
LMT crossover stenting (%)	7 (29)	53 (42)
Contrast dye volume (ml)	166±55.5	156±49.0
Radiation time (min)	32.7±11.2	35.7±17.0
Operation time (min)	97.0±26.7	103±37.4
Recross times	1.38±0.71	1.28±0.52
≥2 recross attempts (%)	6 (25)	31 (25)
3D Guide	5 (21)	67 (53)
Configuration		
Link-free type (%)	6 (25)	78 (62)
Link-connecting type (%)	18 (75)	48 (38)

Values are presented as mean±SD or number (percent); Cr=Creatinine; eGFR=estimated glomerular filtration rate; EF=ejection fraction; AP=angina pectoris; MI=myocardial infarction; ACS=acute coronary syndrome; LMT=left main trunk; LAD=left anterior descending artery; Dx=diagonal branch LCX=left circumflex artery; OM=obtuse marginal; RCA=right coronary artery, PD=posterior desending; PL=posterior lateral; QCA=quantitative coronary angiography; PMV=proximal main vessel; DMV=distal main vessel; SB=side branch.

## 2 Experimental design, materials and methods

Data subjects comprised the 3D-OCT bifurcation registry database (multicenter prospective study: 3 sites with 3D-OCT guided PCI and 7 sites with 2D-OCT guided PCI) and the Nakatsu OCT database (single-center retrospective study, before and after adoption of 3D-OCT reconstruction software). Bifurcation PCI cases meeting the following inclusion and exclusion criteria were extracted from the two databases and analyzed. The inclusion criteria were: (a) angiographically documented bifurcation lesion with ≥75% stenosis of the diameter at least one MV or SB, (b) SB diameter greater than 2.0 mm (visual assessment), (c) treated with crossover stenting followed by KBI under OCT guidance. Exclusion criteria were: (a) side branch <2 mm diameter (visual assessment), (b) in-stent restenosis, (c) congestive heart failure, (d) renal insufficiency with serum creatinine level >1.5 mg/dl except for hemodialysis patients, (e) a two-stent case, and (e) a case in which OCT examination was not performed after GW recrossing (Nagoshi et al., 2018) [1].

## Figures and Tables

**Fig. S1:**
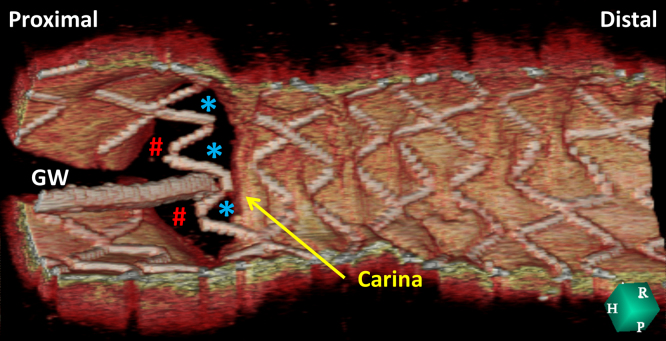
Figure. 1 Proximal and distal cells. The cell between the carina and the adjacent proximal strut is the distal cell. The proximal cell to the distal cell is the proximal cell. (*: Distal cell, #: Proximal cell).

**Fig. S2:**
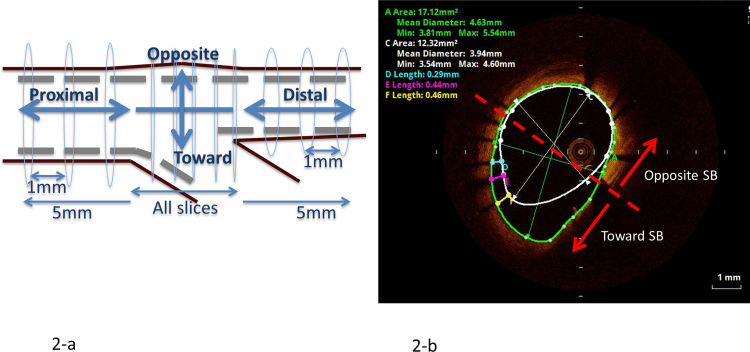
Figure 2 Four analyzed segments and representative figure for calculation of incomplete stent apposition. 2-a; Four analyzed segments; the proximal and distal portions of the MV estimated at every 1-mm, the portions toward and opposite to the SB orifice estimated at every cross-section. 2-b; The struts in the cross section were plotted with white dots. Among the ten dots, the distances between the 3 dots and vessel wall were longer than the stent strut + polymer thickness and the OCT resolution (14 μm) which were accredited as malapposed struts.

**Fig. S3:**
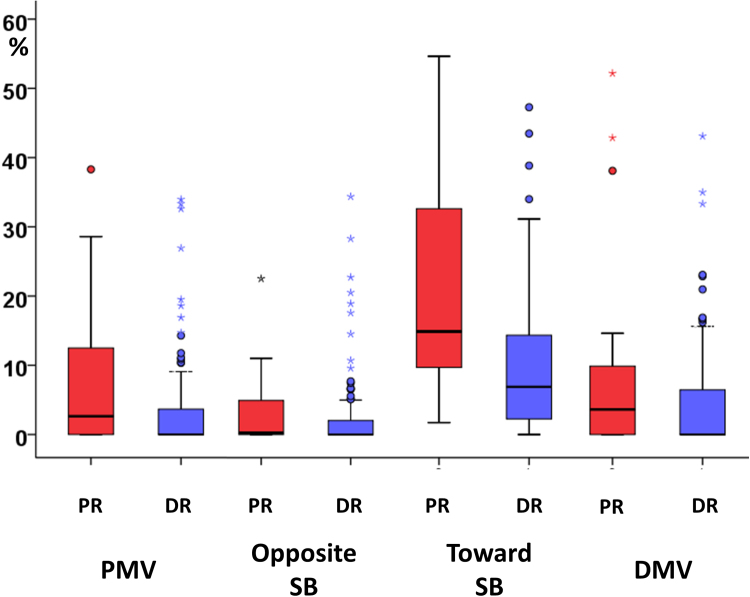
Figure 3 Boxplot for % incomplete stent apposition (%ISA) comparing the proximal and the distal cell recross. Compared with the proximal recross, %ISA in PMV and towards SB are significantly smaller in the distal recross. PMV=proximal main vessel; SB=side branch.

**Fig. S4:**
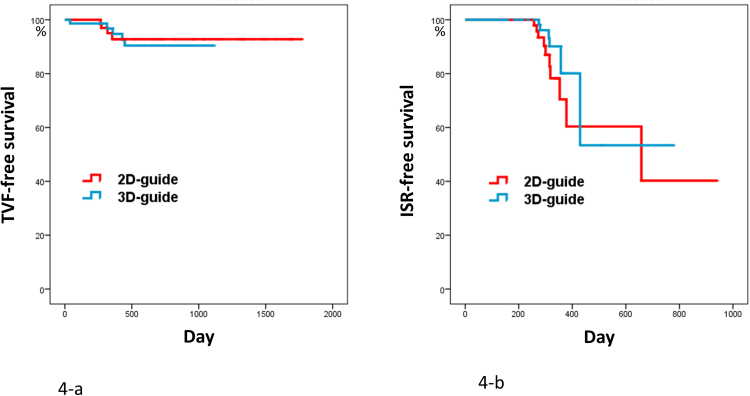
Figure 4 Kaplan-Meier curves of TVR-free and ISR-free survivals under 2D- or 3D-guidance. 4-a; TVF-free survival. 4-b; ISR-free survival. TVF=target vessel failure; ISR=in-stent restenosis.
