# Supplementary material for: Data on two- and three-dimensional optical coherence tomography guidance for the treatment for the bifurcation lesion
Source: Data Brief. 2017 Dec 14;16:865–8. doi: 10.1016/j.dib.2017.12.024 (PMC5847640; doi:10.1016/j.dib.2017.12.024)
Supplement: Supplementary file 1 — Supplementary material [file mmc1.docx]

We wish to draw the attention of the Editor to the following facts which may be considered as potential conflicts of interest and to significant financial contributions to this work.

Dr. Okamura and Dr. Shite received honoraria for technical consulting from St Jude Medical.

The other authors have no disclosure to declare related to this investigation.

We confirm that the manuscript has been read and approved by all named authors and that there are no other persons who satisfied the criteria for authorship but are not listed. We further confirm that the order of authors listed in the manuscript has been approved by all of us.

We confirm that we have given due consideration to the protection of intellectual property associated with this work and that there are no impediments to publication, including the timing of publication, with respect to intellectual property. In so doing we confirm that we have followed the regulations of our institutions concerning intellectual property.

We further confirm that any aspect of the work covered in this manuscript that has involved either experimental animals or human patients has been conducted with the ethical approval of all relevant bodies and that such approvals are acknowledged within the manuscript.

We understand that the Corresponding Author is the sole contact for the Editorial process (including Editorial Manager and direct communications with the office). He is responsible for communicating with the other authors about progress, submissions of revisions and final approval of proofs. We confirm that we have provided a current, correct email address which is accessible by the Corresponding Author (Junya Shite MD, email: [shite@med.kobe-u.ac.jp](mailto:shite@med.kobe-u.ac.jp)).

List of all Authors: Ryoji Nagoshi MD, Takayuki Okamura MD, Yoshinobu Murasato MD, Tatsuhiro Fujimura MD, Masahiro Yamawaki MD, Shiro Ono MD, Takeshi Serikawa MD, Yutaka Hikichi MD, Fumiaki Nakao MD, Tomohiro Sakamoto MD, Toshiro Shinke MD, Yoichi Kijima MD, Amane Kozuki MD, Hiroyuki Shibata MD, Junya Shite MD
